# Prevalence and mortality of chronic kidney disease in lymphoma patients: A large retrospective cohort study

**DOI:** 10.1097/MD.0000000000009615

**Published:** 2018-01-12

**Authors:** Masamitsu Ubukata, Masaki Hara, Yuki Nishizawa, Teruhiro Fujii, Kosaku Nitta, Akihito Ohta

**Affiliations:** a Division of Nephrology, Department of Medicine, Tokyo Metropolitan Komagome Hospital, Bunkyo-Ku; b Department IV^2^, Internal Medicine, Tokyo Women's Medical University, Shinjuku-ku, Tokyo, Japan.

**Keywords:** chronic kidney disease, lymphoma, mortality, prevalence

## Abstract

In patients with lymphoma, an important issue that has been recognized is renal involvement, including glomerulonephritis, acute kidney injury, and lymphoma infiltrating the kidney. However, the prevalence and mortality of chronic kidney disease (CKD) have not been fully understood in lymphoma patients. This study aimed to evaluate the prevalence of CKD and its impact on mortality in those patients.

This was a retrospective cohort study of 429 consecutive lymphoma patients who were admitted or regularly visited our hospital from January 2013 to October 2016. CKD was defined as estimated glomerular filtration rate (eGFR) < 60 mL/min/1.73 m^2^ and/or proteinuria ≥ 1+ that was sustained for at least 3 months. The prevalence of CKD at enrollment was evaluated according to the modified CKD classification by Kidney Disease: Improving Global Outcomes (KDIGO) (eGFR and proteinuria category). Dipstick proteinuria was classified into 3 grades: A1 for − and ±; A2 for 1+ or 2+; and A3 for ≥3+. The eGFR (mL/min/1.73 m^2^) was classified into 6 stages: G1 for ≥90, G2 for 60 to 89, G3a for 45 to 59, G3b for 30 to 44, G4 for 15 to 29, and G5 for <15. The cumulative mortality rate was estimated using the Kaplan–Meier method, with stratification into 2 groups based on the presence or absence of CKD. Furthermore, a multivariate Cox proportional hazards regression model was used to calculate the hazard ratio (HR) and its 95% confidence interval (CI) for all-cause mortality, after adjustments for age, sex, pathologic type, clinical stage of lymphoma, presence or absence of diabetes mellitus, hypertension, and cardiovascular disease.

The mean follow-up period was 3.06 ± 0.96 years, and the prevalence of CKD at study enrollment was 34.5%. The cumulative mortality rate was 20.7%, and was significantly higher in the CKD group than in the group without CKD (36.4% vs 18.0%, *P* = .02). Multivariate analysis found mortality to be significantly associated with CKD (HR 1.58; 95% CI, 1.01–2.46), and this association was the most robust with very high-risk CKD (HR 6.94; 95% CI, 2.50–17.33).

The prevalence of CKD in lymphoma patients was high. CKD should be considered an independent risk factor for mortality among patients with lymphoma.

## 1 Introduction

In the chronic kidney disease (CKD) population, cancer, along with infectious disease and cardiovascular disease (CVD), is recognized as a complication and a major cause of morbidity and mortality. Moderate CKD increases the risk for all cancer in men by approximately 40%, independent of other known risk factors such as age and smoking.^[1]^ Some previous reports have demonstrated that not only does cancer frequently occur in CKD patients, but, conversely, CKD highly frequently occurs in cancer patients.^[2–5]^ The “IRMA” studies in France (Renal Insufficiency and Anticancer Medications) reported that the prevalence of a reduced glomerular filtration rate (GFR) (<90 mL/min/1.73 m^2^) was high, with 52.9% and 50.2% of the patients experiencing this condition in IRMA-1 and IRMA-2, respectively, and 12.0% and 11.8% having stage 3 or higher CKD (GFR < 60 mL/min/1.73 m^2^).^[4,5]^ Also, Nakamura et al^[3]^ reported that the prevalence of CKD, defined as estimated GFR (eGFR) < 60 mL/min/1.73 m^2^, was high, with 25% of subjects experiencing this status in a prospective cohort study of 231 Japanese cancer patients. Furthermore, the survival probability of patients with CKD is significantly decreased compared with patients without CKD.^[3]^ In the IRMA-2 study,^[4]^ the patients with stage 3 or higher CKD at the time of inclusion had a lower survival rate than the patients with GFR ≥ 60 mL/min/1.73 m^2^.

Currently, CKD is reported as an independent risk factor for survival in head and neck, stomach, liver, colorectal, urinary tract, gynecological, and hematologic malignancy.^[6,7]^ Since the impact of CKD on prognosis may differ depending on the type of cancer, each individual site should be considered separately when relationships between mortality and carcinoma are investigated in CKD patients. Other reports have also demonstrated that the presence of CKD was associated with an increased risk of death in cancer patients.^[6–8]^ Iff et al^[8]^ demonstrated that excessive cancer mortality varied with site, with the greatest risk for breast and urinary tract cancer {adjusted hazard ratio (HR) of 1.99 [95% confidence interval (CI), 1.05–3.85; *P* = .01] and 2.54 (95% CI, 1.02–6.44; *P* = .04), respectively}. Iff et al reported that among 370 cancer deaths, the number of lymphoproliferative cancer deaths was 27, and the adjusted HR for mortality was not significant [adjusted HR was 0.62 (95% CI, 0.26–1.46)]. However, the number of cases was small and Iff et al did not investigate lymphoma patients specifically.

As described above, the relationship between CKD and cancer is robust. However, the relationship between lymphoma and the prevalence and mortality of CKD in lymphoma patients has not been fully understood. The aims of our study were to estimate the prevalence of CKD in lymphoma patients and evaluate the mortality risk in lymphoma patients with underlying CKD.

## 2 Materials and methods

### 2.1 Study design

This was a retrospective cohort study in lymphoma patients. Lymphoma was defined according to the 2008 World Health Organization classification criteria. The study was performed in accordance with the Declaration of Helsinki and approved by the institutional committee on research ethics (approval certificate no. 1953).

### 2.2 Study population and data collection

Consecutive data were collected from lymphoma patients who were admitted or regularly visited Tokyo Metropolitan Komagome Hospital in 2013. The inclusion criteria for the present study were as follows: patients with 2 or more visits, follow-up period ≥3 months, and at least 2 consecutive sets of laboratory data.

Demographics and laboratory data that were accumulated in the electronic medical charts were longitudinally followed from January 2013 to October 2016. The following demographic and laboratory data were obtained for all participants: age; sex; the presence of comorbidities including hypertension (HT), diabetes mellitus (DM), and CVD; proteinuria; hemoglobin (Hb); serum concentrations of creatinine (Cr), albumin (Alb), uric acid (UA), C-reactive protein (CRP), and lactate dehydrogenase (LDH); clinical stage; and type of lymphoma. HT was defined as systolic blood pressure ≥140 mm Hg and/or diastolic blood pressure ≥90 mm Hg or the use of antihypertensive agents at baseline. DM was defined according to the World Health Organization diagnostic criteria for diabetes (any of fasting plasma glucose ≥ 126 mg/dL, 2-hour plasma glucose ≥ 200 mg/dL during a 75-g oral glucose tolerance test, HbA1c > 6.5%, and the stipulation that diagnosis of diabetes in an asymptomatic person should not be based on a single abnormal plasma glucose or HbA1c value) or the use of oral antidiabetic agents or insulin at baseline. CVD was defined as angina pectoris, myocardial infarction, other ischemic heart disease (IHD), and heart failure. IHD included angina pectoris and myocardial infarction. Clinical stage was stratified by the Ann Arbor classification. Type of lymphoma consisted of Hodgkin lymphoma (HL) and non-Hodgkin lymphoma (NHL). Further, NHL was classified as follows: mature B-cell lymphoma (diffuse large B-cell lymphoma, follicular lymphoma, MALT lymphoma, mantle cell lymphoma, Burkitt lymphoma, and other B-cell lymphoma), mature T-cell and NK-cell neoplasms, precursor lymphoid neoplasms, and others or unknown.

### 2.3 Measurements and definitions

Proteinuria was defined as ≥1+ on urine dipstick examination. The eGFR was calculated on the basis of serum Cr using the 3-variable Japanese equation constructed by the Japanese Society of Nephrology: eGFR (mL/min/1.73 m^2^) = 194 × serum Cr^−1.094^ × age^−0.287^ × 0.739 (if female). This equation was used because the Modification of Diet in Renal Disease (MDRD) study equation has been shown to be less accurate in Asian patients, including the Japanese.^[9]^

CKD was defined as either eGFR < 60 mL/min/1.73 m^2^ or proteinuria ≥ 1+ or both that were sustained at least for 3 months. Dipstick proteinuria was classified into 3 grades: A1 for − and ±; A2 for 1+ or 2+; and A3 for ≥3+. The eGFR (mL/min/1.73 m^2^) was classified into 6 stages: G1 for ≥90, G2 for 60 to 89, G3a for 45 to 59, G3b for 30 to 44, G4 for 15 to 29, and G5 for <15. The prevalence of CKD at enrollment was evaluated according to the current CKD classification. In brief, the participants were distributed into a 6 × 3 table, combining eGFR with proteinuria (e.g., G3bA3), and the severity of CKD was classified into the following 4 categories: no risk for G1A1 and G2A1; moderate risk for G1A2, G2A2, and G3aA1; high risk for G1A3, G2A3, G3aA2, and G3bA1; and very high risk for G3aA3, G3bA2, G3bA3, G4, and G5.^[10]^

### 2.4 Statistical methods

Data are shown as mean ± standard deviation (SD). Two patient groups were compared using the Mann–Whitney *U* test for continuous variables and the Chi-square test for categorical variables. The cumulative mortality rate over time was estimated using the Kaplan–Meier method, which was stratified into 2 groups by presence or absence of CKD. Statistical significance between 2 groups was compared by the log-rank test. The association of prevalent CKD with mortality over time was analyzed using a Cox multivariate proportional hazards regression model, adjusted for the following known risk factors: age, sex, pathological type (HL, mature B-cell lymphoma, mature T-cell and NK-cell neoplasms, precursor lymphoid neoplasms, and others or unknown), clinical stage of lymphoma, presence or absence of DM, HT, and CVD. All statistical analyses were performed using the statistical software package JMP version 12.0 (SAS Institute Japan, Tokyo, Japan). Values of *P* < .05 were considered significant.

## 3 Results

### 3.1 Demographic and laboratory characteristics of lymphoma patients and prevalence of CKD

In total, 429 patients who were admitted or regularly visited our hospital in 2013 were included. The baseline characteristics of the participants are summarized in Table 1. The mean age of the study population was 60.1 ± 16.6 years, and 253 patients (59.0%) were men. The mean eGFR was 73.7 ± 30.0 mL/min/1.73 m^2^, and the mean serum Cr was 0.84 ± 0.38 mg/dL. Sixty-two patients had proteinuria ≥1+, and 148 patients out of 429 lymphoma patients were diagnosed as having CKD. The prevalence of proteinuria was 14.5% and of CKD was 34.5%. The CKD patients had significantly lower values of Hb, hyperuricemia, and hypoalbuminemia, and higher levels of CRP and LDH than the non-CKD patients. In addition, the CKD patients had a higher prevalence of HT than the patients without CKD (27.7% vs 13.9%; *P* < .001), of DM (24.3% vs 14.9%; *P* = .018). In type of NHL, the CKD patients had the higher prevalence of other B-cell lymphoma (16.9% vs, 7.1%; *P* = .003). Other comorbidities, clinical stage of lymphoma, and type of lymphoma other than B-cell lymphoma did not have a significant difference between the 2 groups.

**Table 1:**
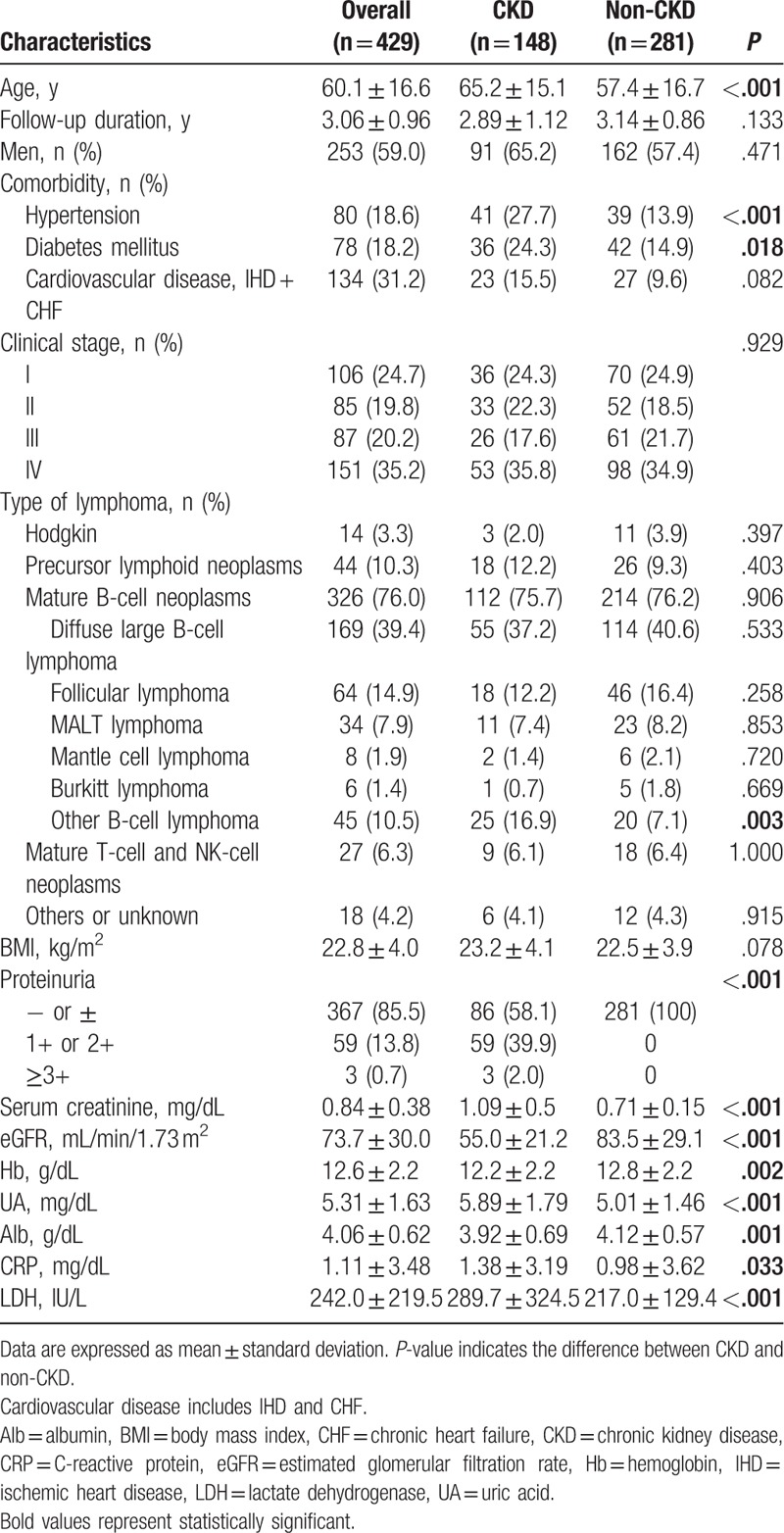
Demographics and laboratory data of lymphoma patients.

The distribution of CKD stratified according to the current CKD classification is shown in Table 2. A total of 114 patients (26.6%) had eGFR < 60 mL/min/1.73 m^2^, 62 patients (14.5%) had proteinuria and 28 patients (6.53%) had both. Therefore, 148 (34.5%) patients were affected by CKD at enrollment. According to this: 281 (65.5%) had no risk (non-CKD; G1A1 and G2A1; white color), 91 (21.2%) had moderate risk (G1A2, G2A2, G3aA1; light gray color), 43 (10.0%) had high risk (G1A3, G2A3, G3aA2, G3bA1; medium gray color), and 14 (3.26%) had very high risk (G3aA3, G3bA2, 3, G4A1-3, G5A1-3; dark gray color). On the other hand, in patients were diagnosed as having CKD, 19 patients improved eGFR more than 30% from baseline during study period.

**Table 2:**
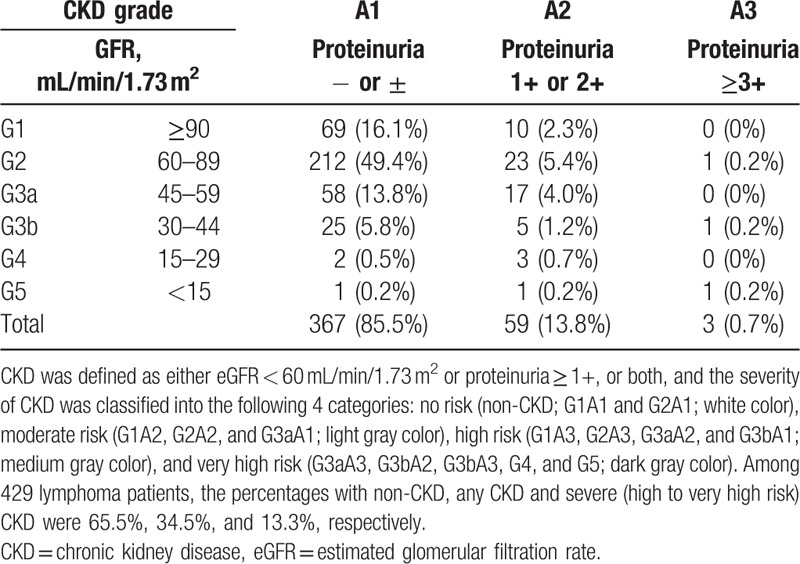
Distribution of CKD in lymphoma.

### 3.2 Influence of CKD on mortality

**Figure 1:**
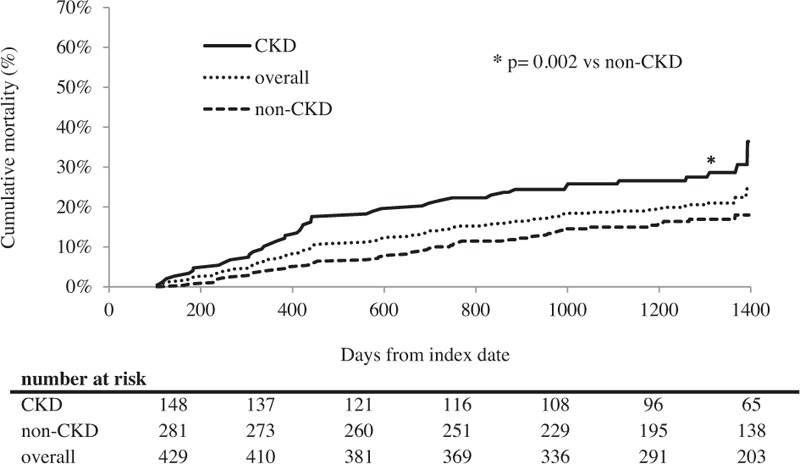
Comparisons of cumulative mortality over time among lymphoma patients. The Kaplan–Meier curves are drawn over 3 years, stratified by the presence or absence of any CKD. The difference between the curves was analyzed with the log-rank test. An asterisk (∗) indicates that the difference between any CKD and non-CKD is significant (*P* = .002). Chronic kidney disease (CKD; solid line), non-CKD (dashed line), and overall patients (dotted line).

During a mean follow-up of 3.06 ± 0.96 years (1312.7 person-years), 89 patients died. All-cause mortality over 3 years was 20.7% (67.80/1000 person-years). There were 43 (29.1%) deaths in the CKD patients and 46 (16.4%) deaths in the non-CKD patients. The Kaplan–Meier curves, stratified by the presence or absence of any CKD, are shown in Fig. 1. The cumulative mortality in CKD patients was significantly higher than in the non-CKD patients (36.4% vs 18.0%; *P* = .02).

**Figure 2:**
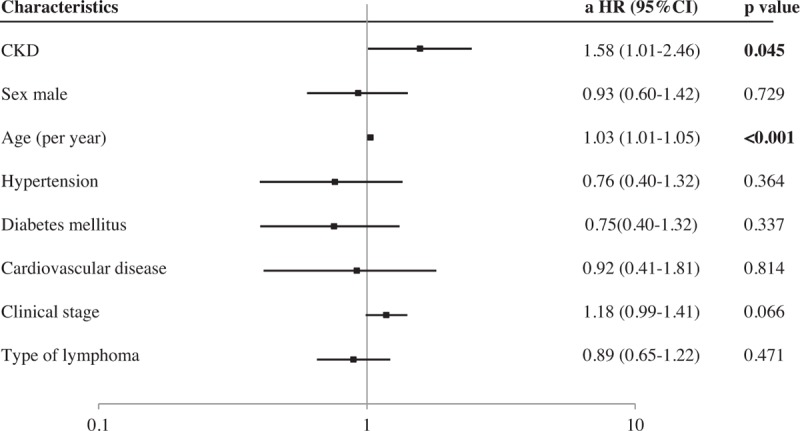
Factors associated with mortality in lymphoma. Multivariate analysis was conducted to identify factors associated with mortality, incorporating the covariates shown in the figure. a-HR = adjusted hazard ratio, CI = confidence interval, CKD = chronic kidney disease.

Table 3 shows the unadjusted hazard ratio (HR) and adjusted hazard ratio (aHR) for all-cause mortality. To test the influence of CKD on all-cause mortality, multivariate Cox proportional hazards regression analysis was conducted, adjusted for the known risk factors of age, sex, pathological type, clinical stage of lymphoma, presence or absence of DM, HT, and CVD. In the unadjusted model, the presence of CKD was significantly associated with mortality [HR 1.93 (95% CI, 1.27–2.92); *P* = .002]. After classification into the 4 categories, moderate risk and very high-risk CKD was found to be significantly associated with mortality [HR 1.87 (95% CI, 1.14–2.99); *P* = .014, HR 4.71 (95% CI, 1.94–9.77); *P* = .002, respectively]. In the adjusted model, the presence of any CKD was also significantly associated with mortality [aHR 1.58 (95% CI, 1.01–2.46); *P* = .045], and only very high-risk CKD was significantly associated with mortality [aHR 6.94 (95% CI, 2.50–17.33); *P* ≤ .001]. Figure 2 demonstrates the association between known risk factors including CKD and mortality that were examined using a multivariate Cox proportional hazards regression model. Higher age [HR 1.03 (95% CI, 1.01–1.05); *P* ≤ .001] were associated with mortality in the lymphoma patients. However, sex, HT, DM, CVD, clinical stage, and types of lymphoma did not have a significant impact on mortality in the lymphoma patients.

**Table 3:**
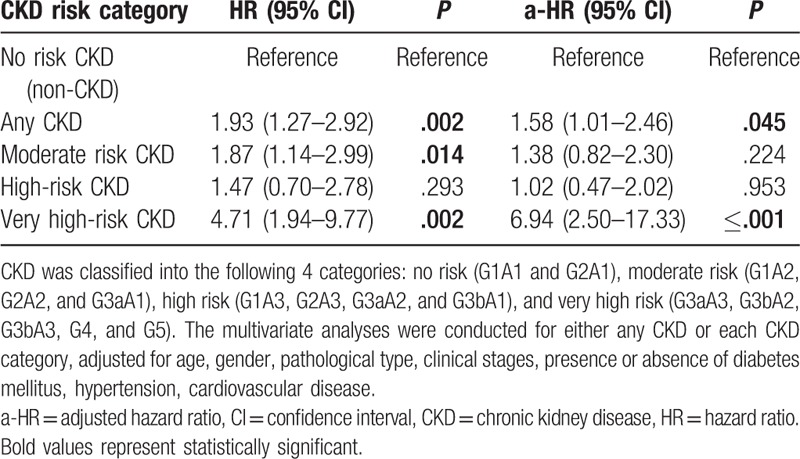
Hazard ratios of CKD for mortality in multivariate models.

## 4 Discussion and conclusions

In this retrospective cohort study of 429 consecutive lymphoma patients who were admitted or regularly visited our hospital from January 2013 to October 2016, there were 148 CKD patients, and the prevalence of CKD was 34.5%. Patients with CKD are associated with increased risk of mortality compared with those without CKD, and this association was the most robust with very high-risk CKD.

In this study, we investigated the prevalence of CKD in lymphoma patients. This is the first study to clarify the prevalence of CKD among lymphoma patients on the basis of combined eGFR and proteinuria data according to the current CKD classification. CKD is a public health problem around the world. Although the incidence and prevalence of CKD vary globally, the prevalence of CKD is consistently reported to be around 11% in high-income countries.^[11]^ In Japanese data obtained for 574,024 (males 240,594; females 333,430) participants over 20 years of age taken from the general adult population, 13% of the Japanese adult population—approximately 13.3 million people—were predicted to have CKD.^[12]^ In the present study, there were 148 CKD patients among 429 lymphoma patients diagnosed according to the current guideline, and the prevalence of CKD was 34.5%. This result indicates that the prevalence of CKD in patients with lymphoma is considerably higher than in that of the general population.

It is reported that albuminuria may reflect a paraneoplastic renal disease and/or may be a marker of cancer incidence and associated mortality.^[13]^ In the present study, 62 (14.5%) of the lymphoma patients had proteinuria. A previous 1-year prospective cohort study^[14]^ was conducted to ascertain the association between proteinuria and mortality in 46 hospitalized NHL patients. The prevalence of proteinuria was 15.2% in the NHL patients, and the cumulative mortality was significantly higher in the group of proteinuric patients than in the nonproteinuric patients. The study showed that serum IL-6 concentration was solely and significantly associated with the presence of proteinuria, and indicated the possibility of systemic inflammation in NHL patients. The prevalence of proteinuria in our study was similar to that of the previous study. Since there is a close association between proteinuria and lymphoma, we should take not only the glomerular filtration rate, but also proteinuria, into consideration. Thus, the current CGA classification appears to be a plausible tool to evaluate CKD in lymphoma patients.

Lymphoma patients with CKD may be at higher risk for mortality than the non-CKD patients. In our study, during a mean follow-up of 3.06 ± 0.96 years (1312.7 person-years), 89 patients died, and all-cause mortality over 3 years was 20.7% (67.80/1000 person-years). The cumulative mortality in the CKD patients was significantly higher than in the non-CKD patients (36.4% vs 18.0%; *P* = .02). Our longitudinal study clarified that CKD was significantly associated with mortality. In the Cox proportional hazards regression analysis after adjusting for known risk factors, CKD was found to be significantly associated with mortality [aHR 1.58 (95% CI, 1.01–2.46); *P* = .045]. Moreover, the moderate risk category and the very high-risk category had an increased likelihood of mortality compared with the no risk category. This suggests that the risk category based on the current CKD classification is of special relevance for predicting the outcomes of lymphoma patients. Indeed, Hong et al^[15]^ reported baseline renal function as a prognostic indicator in patients with newly diagnosed diffuse large B-cell lymphoma. However, the data were inadequate from the point of view of the current CKD concept, as Hong et al showed neither data combining eGFR with proteinuria or albuminuria nor confirmation of the persistence of kidney disease for ≥3 months.

The reason for the strong association between CKD and mortality remains undetermined. Chronic inflammation may explain the association of CKD and oncological outcome.^[16]^ The condition of CKD would be simultaneously associated with immune activation, marked by systemic inflammation and immune deficiency. Systemic inflammation contributes to atherosclerosis, CVD, cachexia, and anemia, while immune deficiency leads to impaired response to vaccination, and increased incidence and severity of microbial infections. Immunocompromised CKD patients may have reduced DNA repair capacity and protection against viral oncogenes. Chronic inflammation and increased oxidative stress underlie uremia-associated immune deficiency.^[17,18]^ Long-term inflammation and oxidative stress caused by CKD and linked to organ degradation may increase carcinogenicity. Frailty could be another potential factor that could be result in a poor prognosis. Patients with CKD are more likely to be frail,^[19]^ and frailty is also one of the important parameters of cancer.^[20,21]^ These factors mentioned above seem to contribute to poor prognosis in lymphoma patients.

Our study had several limitations. First, it was a retrospective analysis, so we could not prove causality. Second, our study was undertaken on the basis that dipstick proteinuria could be utilized as a convenient alternative to albuminuria; therefore, our method differed from the original 2012 Kidney Disease: Improving Global Outcomes (KDIGO) classification in which albuminuria was used. Although albuminuria would be expected to be more accurate for evaluating kidney glomerular damage and prognosis than dipstick proteinuria, the test for albuminuria is more expensive and not readily applicable for use in a general clinical setting from the perspective of the health insurance system in Japan. Furthermore, the causes of CKD were not examined from the pathological point of view. It may result from renal infiltration by lymphoma or toxicity of drugs, etc. However, renal biopsy is generally unfeasible in patients with cancer, especially in undergoing chemotherapies due to the perceived risks. Third, because of the lack of data on performance status (PS) and extranodal lesions, we could not collect information for the International Prognostic Index (IPI), which comprises age ≥60 years, NHL stage III or IV, number of extranodal lesions ≥ 2, PS ≥ 2, and presence or absence of an increase in serum LDH over the reference level of the institutional laboratory. The IPI is used globally for the prediction of overall outcome.^[22]^ Despite its limitations, this was a population-based study conducted in a relatively large lymphoma cohort having adequate data for combining eGFR with proteinuria, which likely allowed for the accurate assessment of CKD in lymphoma.

In conclusion, the prevalence of CKD in lymphoma patients in the present study was 34.5%, which is higher than the rate of 15% among the general population.^[11,12,23]^ Prevalent CKD may be an insidious risk factor linked to increased mortality in lymphoma patients. The present study results suggest that oncologists and nephrologists should be aware of the existence of CKD as one of the critical comorbidities, and may contribute to poor prognosis for lymphoma patients.

## References

[1] Wong G Hayen A Chapman JR Association of CKD and cancer risk in older people. J Am Soc Nephrol 2009;20:1341–50. 19406977 10.1681/ASN.2008090998 PMC2689896

[2] Janus N Launay-Vacher V Byloos E Cancer and renal insufficiency results of the BIRMA study. Br J Cancer 2010;103:1815–21. 21063408 10.1038/sj.bjc.6605979 PMC3008606

[3] Nakamura Y Tsuchiya K Nitta K Prevalence of anemia and chronic kidney disease in cancer patients: clinical significance for 1-year mortality. Nihon Jinzo Gakkai Shi 2011;53:38–45. Japanese. 21370576

[4] Launay-Vacher V Epidemiology of chronic kidney disease in cancer patients: lessons from the IRMA study group. Semin Nephrol 2010;30:548–56. 21146120 10.1016/j.semnephrol.2010.09.003

[5] Launay-Vacher V Oudard S Janus N Prevalence of renal insufficiency in cancer patients and implications for anticancer drug management: the renal insufficiency and anticancer medications (IRMA) study. Cancer 2007;110:1376–84. 17634949 10.1002/cncr.22904

[6] Na SY Sung JY Chang JH Chronic kidney disease in cancer patients: an independent predictor of cancer-specific mortality. Am J Nephrol 2011;33:121–30. 21242672 10.1159/000323740

[7] Yang Y Li HY Zhou Q Renal function and all-cause mortality risk among cancer patients. Medicine (Baltimore) 2016;95:e3728. 27196494 10.1097/MD.0000000000003728 PMC4902436

[8] Iff S Craig JC Turner R Reduced estimated GFR and cancer mortality. Am J Kidney Dis 2014;63:23–30. 23993153 10.1053/j.ajkd.2013.07.008

[9] Matsuo S Imai E Horio M Revised equations for estimated GFR from serum creatinine in Japan. Am J Kidney Dis 2009;53:982–92. 19339088 10.1053/j.ajkd.2008.12.034

[10] Kidney Disease: Improving Global Outcomes (KDIGO) CKD Work Group KDIGO 2012 clinical practice guideline for the evaluation and management of chronic kidney disease. Kidney Int Suppl 2013;3:1–50.

[11] Webster AC Nagler EV Morton RL Chronic kidney disease. Lancet 2017;389:1238–52. 27887750 10.1016/S0140-6736(16)32064-5

[12] Imai E Horio M Watanabe T Prevalence of chronic kidney disease in the Japanese general population. Clin Exp Nephrol 2009;13:621–30. 19513802 10.1007/s10157-009-0199-x

[13] Izzedine H Perazella MA Onco-nephrology: an appraisal of the cancer and chronic kidney disease links. Nephrol Dial Transplant 2015;30:1979–88. 25648910 10.1093/ndt/gfu387 PMC4832985

[14] Hara M Ando M Maeda Y Proteinuria is a simple sign of systemic inflammation that leads to a poor prognosis in individuals affected with non-Hodgkin lymphoma. Clin Nephrol 2014;82:51–7. 24887301 10.5414/CN108132

[15] Hong J Lee S Chun G Baseline renal function as a prognostic indicator in patients with newly diagnosed diffuse large B-cell lymphoma. Blood Res 2016;51:113–21. 27382556 10.5045/br.2016.51.2.113 PMC4931929

[16] Rasool M Ashraf MA Malik A Comparative study of extrapolative factors linked with oxidative injury and anti-inflammatory status in chronic kidney disease patients experiencing cardiovascular distress. PLoS ONE 2017;12:e0171561. 28178330 10.1371/journal.pone.0171561 PMC5298283

[17] Vaziri ND Pahl MV Crum A Effect of uremia on structure and function of immune system. J Ren Nutr 2012;22:149–56. 22200433 10.1053/j.jrn.2011.10.020 PMC3246616

[18] Betjes MG Meijers RW Litjens NH Loss of renal function causes premature aging of the immune system. Blood Purif 2013;36:173–8. 24496187 10.1159/000356084

[19] Chowdhury R Peel NM Krosch M Frailty and chronic kidney disease: a systematic review. Arch Gerontol Geriatr 2017;68:135–42. 27810661 10.1016/j.archger.2016.10.007

[20] Mandelblatt JS Cai L Luta G Frailty and long-term mortality of older breast cancer patients: CALGB 369901 (Alliance). Breast Cancer Res Treat 2017;164:107–17. 28364214 10.1007/s10549-017-4222-8 PMC5479131

[21] Pamoukdjian F Aparicio T Zelek L Impaired mobility, depressed mood, cognitive impairment and polypharmacy are independently associated with disability in older cancer outpatients: the prospective Physical Frailty in Elderly Cancer patients (PF-EC) cohort study. J Geriatr Oncol 2017;8:190–5. 28236586 10.1016/j.jgo.2017.02.003

[22] International Non-Hodgkin's Lymphoma Prognostic Factors Project A predictive model for aggressive non-Hodgkin's lymphoma. N Engl J Med 1993;329:987–94. 8141877 10.1056/NEJM199309303291402

[23] Saran R Robinson B Abbott KC US Renal Data System 2016 Annual Data Report: epidemiology of kidney disease in the United States. Am J Kidney Dis 2017;69 (3 suppl 1):A7–8. 28236831 10.1053/j.ajkd.2016.12.004 PMC6605045

